# Investigation of the Effect of Social Media Addiction on Adults with Depression

**DOI:** 10.3390/healthcare9040450

**Published:** 2021-04-11

**Authors:** Serdar Aydin, Orhan Koçak, Thomas A. Shaw, Betul Buber, Esra Zeynep Akpinar, Mustafa Z. Younis

**Affiliations:** 1 School of Health Sciences, Southern Illinois University Carbondale, 1365 Douglas, Drive, Carbondale, IL 62901, USA; saja@siu.edu; 2 Faculty of Health Science, Istanbul University–Cerrahpasa, 34320 Istanbul, Turkey; orhan.kocak@istanbul.edu.tr; 3 Department of Social Work, Istanbul University–Cerrahpasa, 34320 Istanbul, Turkey; btlbbr96@gmail.com (B.B.); esrazeynep.akpinar@gmail.com (E.Z.A.); 4 College of Health Sciences, Jackson State University, 350 W. Woodrow Wilson Dr, Jackson, MS 39213, USA; mustafa.younis@jsums.edu

**Keywords:** social media, addiction, depression

## Abstract

This study aimed to investigate the effects of social media addiction on depression in adult individuals. For this purpose, the researchers analyzed whether social media dependence had differing impacts according to various variables (age, gender, the highest level of education, duration of daily use of social media, frequency of social media use, etc.). A sample population of 419 people who live in different provinces in Turkey between 18 and 62 years of age participated in the research. The questionnaire form was developed to obtain the Social Media Dependence Scale (SMDS), Beck Depression Inventory scores, and demographic information from the participants. The research was conducted according to the general screening model. Significant differences were found between depression and social media dependency in variables such as the number of children, age, and income. As a result of the study, when social media addiction was examined in terms of gender among socio-demographic variables, no significant difference was found.

## 1. Introduction

Today, with easy accessibility to the internet and technological tools, individuals from many age groups can effectively use social media. The use of social media applications has become an essential part of daily life. Healthy internet use helps individuals use multiple skills, such as reading, writing, selecting, and classifying, while collecting information. On the other hand, uncontrolled internet use may adversely affect the individual’s physical, mental, social, and cognitive development [1]. Behavioral addiction, which is defined as an inability to resist an impulse and an incentive to perform an action that harms the person or others, includes technological addiction types such as internet, smartphone, game, and social media addiction [2]. In 2018, the Internet and Social Media User Stats reported that globally, 42% of the total population used social media, and 51 million people use social media in Turkey, representing 63% of its total population [3]. Additionally, 2.95 billion people globally, and 44 million people (54%) in Turkey, have access to social media only through smartphones. According to the same report, 9 million people are on the internet a day. averaging 7 h, and 48 million people spend about 2 h on social media per day in Turkey. Social media tools such as Facebook and Myspace, which can connect people and allow for sharing thoughts, are preferred by millions of users, and those platforms attract users in various ways [4]. YouTube is the most widely (55%) used social media platform [3].

The terminology of addiction is generally used physiologically in the literature. According to DSM-IV, pathological use/abuse of any substance or stimulant is not defined as… addiction.” Instead, the concept of internet addiction is described as the “problematic/pathological use of the internet” [5]. While in the DSM-IV substance use disorder was broken into two separate diagnoses of substance abuse and substance dependence, in the new revision, DSM-5, they combined these two diagnoses into one, to create a single diagnostic category of substance use disorder. Symptoms of internet addiction, and hence social media addiction, which is a sub-category of internet addiction, can be listed as the following: an increasing and more frequent use of the internet, lying about the amount and duration of the use, constantly having an engaged mind with the internet and its elements, using the internet to avoid problems, and demonstrating continued usage while knowing the consequences of excessive use of the internet. Other behavioral effects consist of physical and mental disorders, increasing stagnation, insomnia, and anger. Accordingly, social effects could result in loss of occupational and leisure activities as well as social isolation [6,7,8].

Increased use of technology and tools like the internet, social media, smartphones, and digital games in daily life may cause addictions with technology that can result in depression. Numerous studies show that these specific addiction types are positively associated with depression [9,10,11,12]. Social media addiction can be defined as a type of psychological dependence that develops through cognitive, sensual, and behavioral processes and produces social, academic, or professional negative results in an individual’s life [13,14]. The DSM-5 underlines it as a common and serios medical illness. Excessive or problematic use of the internet and social media is defined as spending at least 8.5 h per week on it. Spending 21.5 h online per week with internet and social media usage is considered an addiction [15,16]. Internet addiction can lead to serious social and psychological problems and deterioration in individuals’ lives due to excessive and uncontrolled use of social media tools [17]. However, there are various difficulties in identifying the internet’s use as an addiction [7]. Children and young people exist in a world where the internet and social media are readily available, and they have the skill set for using these tools effectively. The researchers on social media addiction have reached the following significant findings in this age group: Individuals born in a world with no internet and then learn how to use it have become addicted to social media at a level far beyond those who were born into the internet [18].

The World Health Organization (WHO) emphasizes addiction, regardless of the kind, as a common mental health disorder in which persistent sadness is accompanied by loss of interest in activities that one usually enjoyed, in addition to a situation that accompanies the inability to perform daily activities for at least two weeks [19]. Social media addiction, being common among children, young people, and adults, can lead to changes in individual and social areas [20]. Ozturk (2002) describes depression as a condition with a deep, sometimes sad, and depressed mood. The depression symptoms are observed in individuals who spend a lot of time on social media and, in particular, in young people [21,22]. The transition from adolescence to adulthood is a period in which the individual is in a state of confusion given the search for identity. In addition to these symptoms, depression is described as a mood in which the individual has feelings and thoughts such as worthlessness, weakness, reluctance, pessimism, or guilt [19,23]. In socio-demographic variables, it is reported that depression occurs in early adulthood (late 20 s) in age-related studies [24].

In addition, internet addiction occurs in individuals with depression because of psychological, physical, and social reasons [25]. As a result, adolescents aged 10 to 19 experience sudden changes in their emotions, thoughts, and social relationships. On the other hand, adults experience social withdrawal, a decrease in interest and activity, and deterioration in friendship relationships [26]. In addition, the lack of social support is significantly related to internet addiction. As such, individuals with depression are more likely to be on the internet for social support [11]. In addition, previous studies demonstrate that the trend for fulfilling the social support need is moving from the traditional way to virtual by using social media platforms, which destroys face-to-face social relationships [27].

## 2. Purpose of the Study

The aim of this study was to investigate the effects of social media addiction on the level of depression in adult individuals. For this purpose, the following research questions were sought:

I. What is the relationship between social media addiction and depression in adults?
II. What variables cause differing levels of addiction in social media use?
III. How are socio-demographic characteristics linked with both social media use and depression?
IV. Which social media platform is causing depression the most in adults?

## 3. Methods

### 3.1. Data

The study population includes all individuals who were over the age of 18 in Turkey at the time of data collection. An age limitation was put on the online system in order to reach those people who are above 18. An online survey resulted in 486 participants living in different communities in Turkey. The data were collected via online questionnaire programs. When eliminating participants who left the questionnaires blank or gave invalid answers, the final number of participants was 419 (*n* = 419).

### 3.2. Data Collection Tools

The Socio-demographic Questionnaire, Social Media Addiction Scale (SMAS), and Beck Depression Inventory (BDI), designed and used in the previous studies for the same purpose of collecting data from the participants, were used in the study. Likert-type scales were utilized in the survey, yielding a reliability of the Social Media Addiction Scale (SMAS) with Cronbach’s alpha coefficient = 0.971.

### 3.3. Socio-Demographic Information Form

In order to determine the socio-demographic characteristics of participants and to measure the effects of these variables on social media addiction and depression levels, a form prepared by the researchers consisting of 17 questions was developed to measure the variables of interest. In addition to the basic demographic characteristics such as participants’ age, gender, educational status, marital status, etc., the researchers aimed to measure variables such as how long participants have been using social media, for what purpose social media had been used, and the duration of daily use of social media.

### 3.4. Social Media Addiction Scale (SMAS)

The Social Media Addiction Scale (SMAS) was developed by Tutgun-Unal and Deniz (2015) to measure the social media addiction of university students [13]. The SMAS consists of 41 items, and scores were obtained from a 5-point Likert scale, which is graded with frequency expressions within the range of “Always (5)”,“Often (4)”,“Sometimes (3)”, “Rarely (2)” to “Never (1)”, indicating that the higher point value indicates more social media addiction [13]. A brief description of the factors that make up SMAS and the items included in these factors are as follows: items 1–12 in the measurement tool are related to the “Busy” dimension and measure the effect of social media in which a person is engaged; items 13–17 are related to the “Emotion and Regulation” dimension and measure social media’s influence on one’s emotions; items 18–22 are related to the “Repetition” dimension and measure the inability to control the use of social media and the repetition of usage of the platform; and items 23–41 are related to the “Conflict” dimension and measure the effect of social media on the negative consequences of a person’s life. The Cronbach alpha value of the scale was found to be highly reliable, with a value of 0.967 [13].

### 3.5. Beck Depression Inventory

The Beck Depression Inventory (BDI), developed by Aron T. Beck in 1961 and revised in 1978 and 1989 [28], is a self-rating scale with 21 items that measures characteristic attitudes and symptoms of depression. In the calculation of the inventory, the lowest score is “0” and the highest score is “3” for each question. Accordingly, the lowest score to be obtained from the inventory is “0” and the highest score is “63”. Based on the scale, a lower result means less depression and a higher result means more depression. The validity and reliability study of the inventory was done by Hisli in 1988, and the Turkish version of translated BDI was used for this study. The cut-off point has been determined as 17 for the Turkish version [28]. The Cronbach’s alpha for the inventory is 0.91. The inventory is designed for adults, and the scoring for the inventory is a 4-point Likert scale.

### 3.6. Data Collection and Analysis

Easy and snowball sampling methods were used as data collection methods. Close and accessibility are preferred. Data collection tools were combined in a single questionnaire and presented to the participants through an online platform. The questionnaire was created on Google Forms, and the link for the survey was distributed to individuals in different cities. Participation took place on a voluntary basis. Total time to complete the survey was about 5–6 min for each participant. Data gathering took place over a span time of 30 days. The total number of participants was 486; however, due to some invalid or missing data, 67 participants were eliminated from the sample group, and 419 participants was the final total sample used.

Upon analysis of the data, it was determined to be distributed normally. Therefore, correlation, independent sample t-test, and One-Way ANOVA analyses were applied to the data, and results were interpreted. Data analysis was performed through the IBM SPSS Statistics 20 package program.

## 4. Results

### 4.1. Socio-Demographic Features of Participants

A total of 29.4% of the participants in the study were men (*n* = 123) and 70.4% were women (*n* = 295). The ages of the participants ranged between 18 and 62, and the average age of the sample was 28.46. The highest level of education of the participants was a follows: 1.4% primary school (*n* = 6), 9.8% secondary education (*n* = 41), 50.1% undergraduate (*n* = 210), and 38.7% graduate (*n* = 162). A total of 77.3% of the participants (*n* = 324) spoke a second language. When looking at their marital status, 66.6% were single (*n* = 279), 31% (*n* = 130) were married, 2.4% (*n* = 10) were divorced/widowed, and 26% (*n* = 109) of participants had children. A total of 10.5% (*n* = 44) of participants lived alone; 47% (*n* = 197) lived with extended family; 30.1% (*n* = 126) lived with their spouse; 12.4% (*n* = 52) lived with a roommate. Considering the employment status of the participants, 42.5% (*n* = 178) were employed; 32.2% (*n* = 135) were students; 21.5% (*n* = 90) were unemployed; 2.9% (*n* = 12) were housewives; and, 1% (*n* = 4) were retired. Income status ranged between 0 and 15,000 with the average income of 2101 Turkish lira.

### 4.2. Information on Participants’ Use of Social Media

A total of 99.5% (*n* = 417) of the participants were using social media, and 52% (*n* = 218) were doing so between one and three hours per day, 20.5% (*n* = 86) between four and six hours per day, 19.3% (*n* = 81) for less than one hour per day, and 8.1% (*n* = 34) for more than seven hours per day. Participants had been using social media for an average of eight years. It was determined that 50.4% (*n* = 211) preferred Instagram, and 23.9% (*n* = 100) preferred Facebook as social media platforms. When considering the objectives of participants’ social media use, 35.1% (*n* = 147) reported using it for getting information, 25.8% (108) for leisure time activities during their free time, and 32.5% (*n* = 136) for entertainment purposes such as online gaming.

### 4.3. Investigation of the Effect of Social Media Addiction on Depression

When the depression levels of the participants were examined, the ratio of those with minimal depression was 39.9%; 29.8% had mild depression; 21.5% had moderate depression; and the rate of those with severe depression was 8.8% (See Table 1).

When the BDI, SMAS and its subscales were statistically analyzed in terms of gender, there was no statistically significant difference between male and female participants in terms of Busyness, Emotion, Repetition, Conflict subscales and Social Media Addiction (*p* > 0.05) (See Table 2).

There was a weak positive correlation between the social media usage time and the subscales: Busyness (*r* = 0.140), Conflict (*r* = 0.119) Emotion (*r* = 0.127); and a weak positive correlation (*r* = 0.143) between SMAS. (See Table 3).

There was a negative correlation between the number of children and the score of Busyness (*p* ** <0.01) (*r* = 0.210); a weak negative correlation between the Emotion score (*p* < 0.05) (*r* = 0.116); a weak negative relationship (*r* = 0.150) between the SMAS (*p* ** < 0.01), as well as a weak negative relationship (*r* = 0.101) between Conflict dimension (*p* * < 0.05) as well (See Table 4).

There was a moderate positive correlation between BDI and Busyness (r = 0.363) and Repetition dimension (r = 0.333) a medium positive relationships between Emotion (r = 0.464); Conflict (r = 0.487); and a positive medium relationship between SMAS (r = 0.484) (See Table 5).

### 4.4. ANOVA Test Results with Multiple Variables

There is a significant relationship between the participants’ ages and SMAS and its sub-scales (*p* < 0.05), as well as with the BDI scores (*p* < 0.05). When the average scores are analyzed, it is seen that the age group with the highest social media addiction has a range of 18–25. There are also significant differences between the ages and depression levels of the participants (*p* < 0.05). Depression scores are higher in the 18–33 age group, while depression scores are lower in the people over 34. Tukey HSD analysis was completed to determine where differences occurred. Those between the ages of 18–25 and those between the ages of 26–33 were found to differ (See Table 6).

As a result of the Anova test applied (Table 7), there was significant difference between the participants’ depression level and marital status (*p* = 0.007 <0.05). When looking at the sub-scales with SMAS, there is a significant difference between the marital status of the participants and the total score of SMAS (*p* = 0.02 <0.05) and the Busyness (*p* = 0.00 <0.05). Tukey HSD analysis was completed to determine for which groups there existed this difference, and it was found that it differs from the participants who are married and those who are single.

There was no significant relationship between participants’ depression, social media addiction, or sub-scales with educational level achieved (*p* > 0.05) (See Table 8).

As a result of the Anova test applied, there is a significant difference between the depression levels and income levels of the participants (*p* = 0.03 <0.05). When looking at the sub-scales with SMAS, there is only a significant difference in the “Busyness” dimension of social media addiction on the participants (*p* = 0.02 < 0.05). As the income levels of the participants increased, their busyness levels decreased (See Table 9).

There is a significant relationship between participants’ working status, depression levels, social media addiction, and its sub-scales (*p* < 0.05). Tukey HSD analysis was completed to determine for which groups there was a difference. We found a difference between employees, students, and job seekers (See Table 10).

Working participants make up the group with the lowest score on the depression scale. The group with the highest score was job seekers and retirees. When social media addictions are compared, the group with the highest level of addiction is, again, job seekers, followed by students.

According to the Anova test result, there is no significant relationship between the depression levels of the participants and the social media platform they use most (*p* = 0.22 > 0.05). There is a significant relationship between the social media addictions of the participants and the most used social media platform (*p* = 0.02 <0.05). It is seen that Instagram users have higher addiction than those using other social media platforms. When looking at the sub-scales, there is no significant difference compared to the social media platform used in the “Repetition” and “Conflict” dimensions (See Table 11).

There is a statistically significant relationship between the time spent by the participants on daily social media and depression, social media addiction, and its sub-scales (*p* = 0.00 <0.05). This indicates that social media addiction and depression increase with the increase in time spent on social media. Tukey HSD analysis was completed to determine for which groups this difference existed, and it was observed that there was a differentiation among all groups (See Table 12).

Based on the Anova test applied, results indicate a significant difference between the participants’ aims of using social media, depression, social media addiction, and its sub-scales (*p* < 0.05). Participants who responded that they used social media to spend their free time had the highest depression rate. The lowest depression score was for people who used social media for information. People who used social media for work had the second-highest depression score. When the SMAS scores were examined, those who used social media to spend their free time had higher addiction scores than those who used it for other purposes. People who used it for information had low SMAS scores as well as depression scores (See Table 13).

## 5. Discussion

This study is an investigation on the relationship between adults’ depression and social media addiction, and the sub-problems mentioned in the aims were analyzed through various statistical methods. In the study, the numerical distribution of the socio-demographic characteristics of the participants was examined. Accordingly, 29.4% of the participants in the study were men (*n* = 123) and 70.4% were women (*n* = 295). When the effect of social media addiction and its sub-scales on depression was examined, there was no statistically significant difference between male and female participants in terms of Busyness, Emotion, Repetition, Conflict subscales, and Social Media Addiction (*p* > 0.05). In a study conducted by Kirik et al. [29] on social media addiction with 271 undergraduates, no significant difference was found in terms of gender either. There are also studies in the literature showing that social media addiction of men is higher than in women [30,31,32]. Participants in the current study were using social media for an average of eight years. As a result of the correlation analysis, it was determined that there was a positive weak correlation with SMAS total score and the Busyness and Conflict sub-dimensions (*p* < 0.05). In line with these results, it can be said that the participants who were using social media for a long time had high social media addiction scores and that social media had a large place in their daily lives and negatively affected their lives. It was noted that 26% (*n* = 109) of the participants in the study have children, and the number of their children is between one and nine.

As a result of the correlation test, it was seen that there is a negative relationship between the number of children a participant has and Busyness, Emotion, Conflict sub-scales, and social media addiction (*p* <0.05). Accordingly, it is possible to say that as the number of children increases, social media addiction decreases. Since this situation increases the responsibilities of the mother or father who cares for the child, there will be a direct decrease in the time allocated to social media and social media that is prevented from creating a negative impact on the individual’s life. There is a positive relationship between depression and social media addiction and the Business, Emotion, Repetition, and Conflict sub-scales. Accordingly, when social media addiction, Occupation, Emotion, Repetition, and Conflict sub-scales increase, the level of depression will increase in parallel. Studies in the literature indicate that as the level of internet usage and addiction increases, depression increases [11,33].

There is a significant relationship between the participants’ age group and SMAS and its sub-scales (*p* < 0.05). This result overlaps with the results obtained by Kirik et al. [29]. As a result of the Anova test, it was found there was a significant difference between the participants’ depression level and marital status (*p* < 0.007). When looking at the sub-scales with SMAS, there was a significant difference between the marital status of the participants and the SMAS total score (*p* < 0.05) and the Busyness sub-scale. In this respect, it could be said that married individuals have less time to spend than single individuals in social media because of the increase in their family responsibilities.

There is a significant relationship between participants’ working status, depression levels, social media addiction, and its sub-scales (*p* < 0.05). According to the results, it could be argued that individuals who work spend less time on social media than students or job seekers, and it varies depending on the job conditions. The results also indicate the retiree group has the least tendency toward online addiction, scoring the lowest for the total SMAS total score, Busyness, and Conflict, yet they had the highest BDI score. Besides, it was determined that 50.4% (*n* = 211) of participants preferred Instagram and 23.9% (n = 100) preferred Facebook.

Additionally, there is a significant relationship between the social media addictions of the participants and the most used social media platform (*p* < 0.05). Instagram users were found to have higher dependence than those using other social media platforms. In parallel with this result, according to Turan’s [34] study on undergraduates, a statistically significant difference was found between the students’ internet addiction scale score and the most used social media platforms. A total of 52% of the participants (*n* = 218) spent one to three hours a day on social media.

In addition, there is a statistically significant relationship between the time spent by the participants on daily social media and depression, social media addiction, and its sub-scales (*p* < 0.05). This indicates that social media addiction and depression increase as daily social media usage time increases. In other words, it can be said that internet usage time increases social media addiction. Conversely, participants used more daily internet due to their social media addiction. This addiction-related result overlaps with the results obtained by Kirik et al. [29].

In a study conducted by Balci and Ayhan [35], the factors affecting internet use of undergraduates are specified as a social escape, information acquisition, recreation, economic benefit, social interaction, and entertainment. Another study conducted by Altayef [32] found a significant difference only in the course preparation dimension of the scale of the social media usage objectives and stated that, as the age decreases, the students used social media more for the preparation of lessons. According to a study conducted by Ak et al. [36], there is a significant relationship between the use of social media and online gaming and the level of internet addiction. In conclusion, the risk of being internet addicted is significantly related to the use of social media.

## 6. Conclusions

Increased use of technology and tools like the internet, social media, smartphones, and digital games in daily life has a crucial role in addictions with technology that can result in depression. This study proves that those who were using social media for a long time had high social media addiction scores. In other words, social media had a large place in their daily lives and negatively affected their lives. With the internet becoming an indispensable part of human life, social media addiction is likely to increase gradually. Excessive use of social media tools poses various risks in terms of psychological, physical, and social aspects, in addition to causing problems in the social functionality of individuals. The group most at risk to experience social media addiction are adolescents. It is a necessary to consider differential health policies in relating to the functional change of the “digital age”. Taking into consideration the changing social problems of public policies, it is useful to prepare action plans for behavioral addiction types such as social media addiction, game addiction, and internet addiction, which have been frequently encountered in recent years. It will be necessary to implement such action plans as soon as possible. In order to facilitate the diagnosis and treatment processes of these types of behavioral addictions, relevant rehabilitation and diagnosis centers should be established. At the same time, the lack of social support and the lack of social environment in the orientation of individuals to social media tools causes such addictions. Therefore, family group studies, family therapy, and seminars should be organized to strengthen family dynamics.

## Figures and Tables

**Table 1:** Participants’ Level of Depression.

Beck Depression Inventory Levels	N	Percentage
Minimal Depression (0–9)	167	39.9
Mild Depression (10–16)	125	29.8
Moderate Depression (17–29)	90	21.5
Severe Depression (30–63)	37	8.8

**Table 2:** Investigation of depression and social media addiction subscales in terms of gender (t-Test).

Variables	Gender	n	Average	Ss	t	*p*
BDI Score	Man	123	12.504	10.635	1.346	0.180
	Woman	295	14.040	10.646		
SMAS Total Score	Man	123	79.390	31.545	2.717	0.007
	Woman	295	88.633	32.068		
Busyness	Man	123	32.647	10.594	3.713	0.180
	Woman	295	28.349	11.227		
Emotion	Man	123	20.569	5.333	2.743	0.007
	Woman	295	23.308	9.400		
Repetition	Man	123	8.926	5.333	2.339	0.020
	Woman	295	10.223	5.094		
Conflict	Man	123	30.167	14.368	1.446	0.150
	Woman	295	32.857	14.589		

BDI Scores: Lowest depression = 0, Highest depression = 63.

**Table 3:** Investigation of SMAS and Its Sub-scales in Terms of Social Media Usage Time (Years).

		Busyness	Emotion	Repetition	Conflict	SMAS	BD
Usage Time (year)	*r*	0.140 **	0.127 **	0.059	0.119 *	0.143 **	0.088
	*p*	0.004	0.009	0.225	0.015	0.003	0.074
	n	418	418	418	418	418	418

*p* * < 0.05; *p* ** < 0.01.

**Table 4:** Investigation of the Relationship between SMAS, its Sub-scales, and Number of Children.

		Busyness	Emotion	Repetition	Conflict	SMAS	BD
Number of Children	*r*	−0.210 **	−0.116 *	−0.091	−0.101 *	−0.150 **	−0.091
	*p*	0.000	0.018	0.064	0.040	0.002	0.064
	n	418	418	418	418	418	418

*p* ** < 0.01 *p* * < 0.05.

**Table 5:** Investigation of the Relationship Between Depression Inventory and SMAS and its Sub-scales.

		Busyness	Emotion	Repetition	Conflict	SMAS
BDI	r	0.363 ^**^	0.464 ^**^	0.335 ^**^	0.483 ^**^	0.484 ^**^
	*p*	0.000	0.000	0.000	0.000	0.000
	n	419	419	419	419	419

*p* ** < 0.01.

**Table 6:** Investigation of Depression and SMAS and its Sub-scales in terms of Age (ANOVA).

Variables	Age	*n*	Average	Ss	F	*p*
1BDI	18–25	197	14.3	11	3.39	0.01 *
	26–33	138	14.4	10.4		
	34–41	78	10.2	8.9		
	Over 42	6	11.1	14.5		
SMAS Total Score	18–25	197	92	32.7	8.04	0.00 *
	26–33	138	85.9	31.4		
	34–41	78	72.5	27.4		
	Over 42	6	65.6	28.7		
Busyness	18–25	197	33.9	11.1	10.75	0.00 *
	26–33	138	31	10.6		
	34–41	78	26.1	10.0		
	Over 42	6	23.8	12.9		
Emotion	18–25	197	23.9	9.5	6.12	0.00 *
	26–33	138	22.7	9.1		
	34–41	78	18.8	8.7		
	Over 42	6	18.6	9.7		
Repetition	18–25	197	10.6	5.5	4.59	0.003 *
	26–33	138	9.8	5.2		
	34–41	78	8.2	4.4		
	Over 42	6	6.8	4		
Conflict	18–25	197	34.3	15.4	4.99	0.002 *
	26–33	138	32.2	14.5		
	34–41	78	27.5	10.7		
	Over 42	6	23.1	7.4		

* *p* < 0.05.

**Table 7:** Investigation of Depression and SMAS and its Sub-scales in Terms of Marital Status (ANOVA).

Variables	Marital Status	*n*	Average	ss	F	*p*
SMAS Total Score	Single	279	80.49	30.2	2.90	0.002 *
	Married	130	80.38	24.8		
	Divorced/Widowed	10	91.21	33.7		
		419	81.64	31.8		
BDI	Single	279	14.34	0.64	5.01	0.007 *
	Married	130	11.46	0.86		
	Divorced/Widowed	10	19.70	3.58		
		419	13.57	0.51		
Busyness	Single	279	33.11	10.97	10.26	0.000 *
	Married	130	27.84	10.87		
	Divorced/Widowed	10	30.40	12.13		
		419	31.41	11.21		
Emotion	Single	279	23.44	9.34	4.59	0.011 *
	Married	130	20.46	9.30		
	Divorced/Widowed	10	23.70	10.22		
		419	22.52	9.43		
Repetition	Single	279	10.22	5.33	2.037	0.013 *
	Married	130	9.10	5.17		
	Divorced/Widowed	10	9.50	5.10		
		419	9.85	5.29		
Conflict	Single	279	30.62	13.4	3.96	0.020 *
	Married	130	29.65	10.5		
	Divorced/Widowed	10	33.38	15.5		
		419	33.01	15.3		

* *p* < 0.05.

**Table 8:** Investigation of Depression and SMAS and its Sub-scales in Terms of Educational Levels Achieved (ANOVA).

Variables	Educational Status	*n*	Average	ss	F	*p*
BDI	Primary school	6	15.16	18.8	0.69	0.55
	High School and Equivalent	41	15.2	13.5		
	Associate Degree/Undergraduate	210	13.7	10.6		
	Graduate (Master or Ph.D.)	162	12.8	9.4		
SMAS Total Score	Primary school	6	90.5	51.9	0.21	0.88
	High School and Equivalent	41	88.7	33.7		
	Associate Degree/Undergraduate	210	85.0	32.2		
	Graduate (Master or Ph.D.)	162	86.4	31.1		
Busyness	Primary school	6	30.8	18.1	0.54	0.64
	High School and Equivalent	41	30.8	11.7		
	Associate Degree/Undergraduate	210	30.8	11.2		
	Graduate (Master or Ph.D.)	162	32.3	10.8		
Emotion	Primary school	6	24.8	14.3	0.25	0.85
	High School and Equivalent	41	23.1	10.3		
	Associate Degree/Undergraduate	210	22.6	9.5		
	Graduate (Master or Ph.D.)	162	22.1	8.9		
Repetition	Primary school	6	10.0	7.5	0.01	0.99
	High School and Equivalent	41	9.78	5.8		
	Associate Degree/Undergraduate	210	9.82	5.3		
	Graduate (Master or Ph.D.)	162	9.91	5.02		
Conflict	Primary school	6	34.8	21.3	0.53	0.65
	High School and Equivalent	41	34.7	13.5		
	Associate Degree/Undergraduate	210	31.7	14.5		
	Graduate (Master or Ph.D.)	162	32.0	14.5		

**Table 9:** Investigation of Depression and SMAS and its Sub-scales in Terms of Participants’ Income Levels (ANOVA).

Variables	Income	*n*	Average	ss	F	*p*
BDI Score	0–2000	247	14.63	11.2	2.867	0.036
	2100–4500	118	12.77	10.7		
	4600–6500	36	11.41	5.1		
	7000–15,000	15	8.13	8.2		
SMAS Total Score	0–2000	247	89.29	32.6	2.450	0.063
	2100–4500	118	82.80	33.4		
	4600–6500	36	79.19	25.3		
	7000–15,000	15	74.46	23.4		
Busyness	0–2000	247	32.61	10.9	3.284	0.020
	2100–4500	118	30.03	11.8		
	4600–6500	36	29.77	10.9		
	7000–15,000	15	25.40	7.8		
Emotion	0–2000	247	23.13	9.5	1.249	0.291
	2100–4500	118	21.90	9.9		
	4600–6500	36	21.50	7.8		
	7000–15,000	15	19.26	6.9		
Repetition	0–2000	247	10.23	5.4	1.764	0.153
	2100–4500	118	9.63	5.3		
	4600–6500	36	8.69	4.5		
	7000–15,000(	15	7.86	2.7		
Conflict	0–2000	247	33.62	15.2	2.304	0.076
	2100–4500	118	30.87	14.5		
	4600–6500	36	27.91	9.5		
	7000–15,000	15	29.80	11.1		

**Table 10:** Investigation of Relationship of Participants’ Employment Status with Depression, SMAS and its Sub-scales (ANOVA).

Variables	Employment Status	*n*	Average	ss	F	*p*
BDI Score	Employee	178	11.38	9.20	6.366	0.00 *
	Student	135	13.38	10.18		
	Housewife	12	13.16	11.74		
	Retiree	4	18.2	14.99		
	Jobseeker	90	18.04	12.32		
SMAS Total Score	Employee	178	79.92	30.11	3.811	0.004 *
	Student	135	90.58	31.71		
	Housewife	12	76.08	33.2		
	Retire	4	75.25	37.65		
	Jobseeker	90	92.92	34.4		
Busyness	Employee	178	29.18	11.17	4.525	0.001 *
	Student	135	33.41	10.40		
	Housewife	12	27.16	12.58		
	Retiree	4	26.0	9.66		
	Jobseeker	90	33.63	11.46		
Emotion	Employee	178	21.12	9.05	2.401	0.04 *
	Student	135	23.50	9.70		
	Housewife	12	19.66	9.55		
	Retiree	4	22.0	16.39		
	Jobseeker	90	24.24	9.13		
Repetition	Employee	178	9.00	4.79	3.03	0.01 *
	Student	135	10.79	5.71		
	Housewife	12	7.75	5.25		
	Retiree	4	9.25	7.84		
	Jobseeker	90	10.45	5.24		
Conflict	Employee	178	29.62	12.75	3.194	0.01 *
	Student	135	34.18	14.68		
	Housewife	12	29.25	13.01		
	Retiree	4	27.25	12.01		
	Jobseeker	90	35.04	16.98		

* *p*< 0.05.

**Table 11:** Investigation of Depression and SMAS and its Sub-scales on the Most Common Social Media Platforms (ANOVA).

Variables	The Most Common Platform	*n*	Average	ss	F	*p*
BDI Score	Facebook	100	13.73	11.77	1.43	0.22
	Twitter	44	11.68	10.02		
	Instagram	211	14.57	10.51		
	Youtube	59	11.49	9.46		
	Other	3	10.66	9.8		
SMAS Total Score	Facebook	100	80.49	30.2	2.90	0.02 *
	Twitter	44	80.38	24.8		
	Instagram	211	91.21	33.7		
	Youtube	59	81.64	31.8		
	Other	3	73.33	27.3		
Busyness	Facebook	100	28.34	10.1	7.25	0.00 *
	Twitter	44	29.63	9.5		
	Instagram	211	34.21	11.6		
	Youtube	59	28.16	9.9		
	Other	3	27.33	12.6		
Emotion	Facebook	100	21.53	9.1	2.72	0.02 *
	Twitter	44	21.09	7.9		
	Instagram	211	23.95	9.6		
	Youtube	59	20.45	9.3		
	Other	3	17.33	6.4		
Repetition	Facebook	100	9.57	4.9	1.10	0.35
	Twitter	44	9.68	5.07		
	Instagram	211	10.31	5.5		
	Youtube	59	8.98	5.3		
	Other	3	7.00	2.6		
Conflict	Facebook	100	33.38	13.4	1.06	0.37
	Twitter	44	30.62	10.5		
	Instagram	211	29.65	15.5		
	Youtube	59	33.01	15.3		
	Other	3	28.66	9.5		

* *p* < 0.05

**Table 12:** Investigation of Depression and SMAS and its Sub-scales in Terms of Daily Time on Social Media (ANOVA).

Variables	Daily Time on Social Media (hr)	*n*	Average	ss	F	*p*
BDI Score	Less than 1	81	10.64	10.6	11.5	0.00 *
	1–3	218	12.17	9.5		
	4–6	86	17.62	11.2		
	7 or more	34	19.29	11.3		
SMAS Total Score	Less than 1	81	62.85	19.6	50.2	0.00 *
	1–3	218	81.97	27.8		
	4–6	86	104.79	28.6		
	7 or more	34	119.41	38.2		
Busyness	Less than 1	81	21.85	7.7	69.0	0.00 *
	1–3	218	30.13	9.3		
	4–6	86	38.95	9.2		
	7 or more	34	43.35	11.01		
Emotion	Less than 1	81	16.48	6.4	37.0	0.00 *
	1–3	218	21.38	8.5		
	4–6	86	28.25	8.8		
	7 or more	34	29.79	10.01		
Repetition	Less than 1	81	6.98	3.1	19.3	0.00 *
	1–3	218	9.57	4.9		
	4–6	86	12	5.6		
	7 or more	34	13.11	6.6		
Conflict	Less than 1	81	24.53	7.9	28.6	0.00 *
	1–3	218	30.54	12.7		
	4–6	86	38.18	15.06		
	7 or more	34	46.26	20.2		

* *p* < 0.05

**Table 13:** Investigation of Depression and SMAS and its Sub-scales in Terms of The Purpose of Social Media Use.

Variables	The Purpose of Social Media Use	*n*	Average	ss	F	*p*
BDI Score	To share my activities	18	12.3	9.7	4.8	0.00 *
	For entertainment purposes	136	14.3	10.9		
	For information	147	10.7	8.69		
	To spend my free time	108	16.3	12.01		
	For my business	10	15.4	10.10		
SMAS Total Score	To share my activities	18	81.6	36.3	7.83	0.00 *
	For entertainment purposes	136	91.8	35.4		
	For information	147	75.1	25.7		
	To spend my free time	108	94.5	31.6		
	For my business	10	80.2	26.3		
Busyness	To share my activities	18	30.3	12.1	10.24	0.00 *
	For entertainment purposes	136	33.5	11.4		
	For information	147	27.1	9.51		
	To spend my free time	108	34.9	11.2		
	For my business	10	28.8	8.5		
Emotion	To share my activities.	18	21.8	11.2	5.96	0.00 *
	For entertainment purposes.	136	23.7	10		
	For information.	147	19.7	8.05		
	To spend my free time.	108	24.9	9.2		
	For my business.	10	21.9	10.3		
Repetition	To share my activities.	18	9.9	5.6	2.30	0.05
	For entertainment purposes	136	10.4	5.6		
	For information	147	8.8	4.5		
	To spend my free time	108	10.5	5.6		
	For my business	10	9.6	5.08		
Conflict	To share my activities.	18	29.3	14.5	5.14	0.00 *
	For entertainment purposes	136	34.7	17.1		
	For information	147	28.3	10.6		
	To spend my free time	108	35.1	14.7		
	For my business	10	29.5	9.6		

* *p* < 0.05.

## Data Availability

The data presented in this study are available on request from the corresponding author. The data are not publicly available due to privacy and ethical concerns.
